# Supplementary material for: Fission yeast Swi2 designates cell-type specific donor and stimulates Rad51-driven strand exchange for mating-type switching
Source: Nucleic Acids Res. 2023 Mar 23;51(8):3869–87. doi: 10.1093/nar/gkad204 (PMC10164574; doi:10.1093/nar/gkad204)
Supplement: gkad204_Supplemental_File [file gkad204_supplemental_file.pdf]

Supplementary Materials For

Fission yeast Swi2 designates cell-type specific donor and stimulates Rad51-driven strand exchange for mating-type switching

By

Takahisa Maki, Geneviève Thon, Hiroshi Iwasaki

Supplementary Materials including
Supplementary Tables S1 and S2
Supplementary Figures S1- S3

Supplementary Table S1. List of Strains used in the study

Strain (*S. pombe*)	Mating type region	Other
EM20	h^{90}	*leu1-32 his3-D1 ade6-M375*
TM366	h^{90}	*swi2-5A leu1-32 his3-D1*
TM511	h^{90}	*swi2-AT1-AA leu1-32 his3-D1*
TM519	h^{90}	*swi2-AT2-AA leu1-32 his3-D1*
TM527	h^{90}	*swi2-AT12-AA leu1-32 his3-D1*
TM360	h^{90}	*(Blp1)::LEU2 swi6D::ura4 ura4-D18 leu1-32 ade6-216*
TM689	h^{90}	*swi2-5A-9PK::kamR lue1-32, his3-D1*
TM880	h^{90}	*swi6D::LEU2 swi2-5A-9PK::kamR leu1-32 his3-D1*
M693	h^{90}	*swi2-AT12-9PK::kamR lue1-32, his3-D1*
TM875	h^{90}	*swi6D::LEU2 swi2-AT12-9PK::kamR leu1-32 his3-D1*
TM108	*mat1-PΔ17::LEU2*	*swi2-9PK::kamR*
TM107	*mat1-Msmt-0*	*swi2-9PK::kamR*
TM705	*mat1-PΔ17::LEU2*	*swi2-5A-9PK::kamR ura4-D18 leu1-32*
TM711	*mat1-Msmt-0*	*swi2-5A-9PK::kamR ura4-D18 leu1-32*
TM709	*mat1-PΔ17::LEU2*	*swi2-AT12-AA-9PK::kamR ura4-D18 leu1-32*
TM714	*mat1-Msmt-0*	*swi2-AT12-AA-9PK::kamR ura4-D18 leu1-32*
TP75	*mat3-M SRE3Δ*	*ura4-D18 leu1-32 ade6-M210*
TM857	*mat3-M SRE3Δ*	*swi2-5A-9PK::kamR*
TM802	*mat3-M SRE3Δ*	*swi2-AT12-AA-9PK::kamR*
TP8	*mat2-P SRE2Δ*	*ura4-D18 leu1-32 ade6-M210*
TM797	*mat2-P SRE2Δ*	*swi2-5A-9PK::kamR*
TM852	*mat2-P SRE2Δ*	*swi2-AT12-AA-9PK::kamR*
NO47	h^{90}	*set1D:: kamR*
TM867	h^{90}	*set1D::hphR swi2-5A-9PK::kanR leu1-32 his3-D1 ura4-D18*
TM868	h^{90}	*set1D::hphR swi2-AT12-AA-9PK::kanR leu1-32 his3-D1 ura4-D18*
TM19	h^{90}	*swi2D::arg3 ura4-D18 leu1-32 his3-D1 arg3-D1*
TM100	*mat1-Msmt-0*	
TM534	h^{90}	*swi2-3A eu1-32 his3-D1 ade6-M375*

Strain (*S. cerevisiae*)	Mating type	Others
AH109	*MATa*	*rp1-901, leu2-3, 112 ura3-52 his3-200 gal4Δ gal80Δ* $LYS2::GAL1_{UAS}$-$GAL1_{TATA}$-*HIS3 MEL1* $GAL2_{UAS}$-$GAL2_{TATA}$-*ADE2* $URA3::MEL1_{UAS}$-$MEL1_{TATA}$-*lacZ*

Table S2. List of Oligonucleotides used in this study

Target gene and used plasmid names	Oligonucleotide Sequence from 5'to 3'
**Yeast two hybrid**	
pGADT7-Swi2_Forward	TAATATCCCGGGTGGGCATCG
Swi2 (1-280)_Reverse	CCACCCGGGATATTAATATGGTAACCGGAATGAC
Swi2 (1-260)_Reverse	CCACCCGGGATATTAACAGAGTAGGATATATTTCC
Swi2-F271A_Forward	ACCGAGAAAAGTTGCCGATGCTGTAGTC
Swi2-F271A Reverse	GACTACAGCATCGGCAACTTTTCTCGGT
Swi2-D272A_Forward	GAAAAGTTTTCGCTGCTGTAGTCATTCCG
Swi2-D272A Reverse	CGGAATGACTACAGCAGCGAAAACTTTTC
Swi2-V274A_Forward	CGATGCTGCAGTCATTCCGGTTACC
Swi2-V274A Reverse	GGTAACCGGAATGACTGCAGCATCG
Swi2-V275A_Forward	CGATGCTGTAGCCATTCCGGTTACCAT
Swi2-V275A Reverse	ATGGTAACCGGAATGGCTACAGCATCG
Swi2-I276A_Forward	CGATGCTGTAGTCGCTCCGGTTACCAT
Swi2-I276A Reverse	ATGGTAACCGGAGCGACTACAGCATCG
Swi2-P277A_Forward	GCTGTAGTCATTGCGGTTACCATAAAAC
Swi2-P277A Reverse	GTTTTATGGTAACCGCAATGACTACAGC
Swi2-V278A_Forward	GTAGTCATTCCGGCTACCATAAAACCTAG
Swi2-V278A Reverse	CTAGGTTTTATGGTAGCCGGAATGACTAC
Swi2-T279A_Forward	AGTCATTCCGGTTGCCATAAAACCTAGTAT
Swi2-T279A Reverse	ATACTAGGTTTTATGGCAACCGGAATGACT
Swi2 5A_Forward	GGATTAAGAAACCGAGAAAAGTTGCCGCTGCTGCAGCCGCTCC GGTTACCATAAAACC
Swi2 5A_Reverse	GGTTTTATGGTAACCGGAGCGGCTGCAGCAGCGGCAACTTTTCT CGGTTTCTTAATCC
Swi2 (1-280)_Reverse	CGCCATGGCCAGTTATAAGCGGGTTCGCGAAATC
Swi2 (1-560)_Reverse	CGCCATGGCCAGTTATACAGACATTTTATTAACATTC
Swi2-3A_Forward	CGAAAGGCTCTCATTGCAGCAGCTCATCCTCCGATTTCGAAG
Swi2-3A_Reverse	CTTCGAAATCGGAGGATGAGCTGCTGCAATGAGAGCCTTTCG
**Strain construction**	
F_ Swi2-5A_ gRNA	TCGATGCTGTAGTCATTC
R_ Swi2-5A_ gRNA	GAATGACTACAGCATCGA
F_Swi2-AT1-A gRNA	GAGTGAAGCATAGGGCAGGA
R_ Swi2-AT1-A gRNA	TCCTGCCCTATGCTTCACTC
F_Swi2-AT2-A or -AT12-AA gRNA	GAAGGATTGCCAAGAAGAAG
R_ Swi2-AT1-A or -AT12-AA gRNA	CTTCTTCTTGGCAATCCTTC
F_ Swi2-3A gRNA	TTCGAAATCGGAGGATGAAA
R_ Swi2-3A gRNA	TTTCATCCTCCGATTTCGAA
F_Swi2-5A	GGGGAAGACCTACAGGCTGG
R_Swi2-5A	CAGGGAAGTCTTCATCCTCTCTTG

F_Swi2-AT1-A, AT2-A or AT12-AA	GGTTGGAACCAGCTGTGTG
R_ Swi2-AT1-A, AT2-A or AT12-AA	TGAGGTAACGAGGGCTCTG
F_Swi2-3A	GATAAATCGAAGCCTTCCATCTC
R_ Swi2-3A	GCCACCAATTTCTAATTCAAGGT
F_Swi2-9V5	CATTGGTACGTACCTTGAACAGTTGCAAATACCCTTCCATCTCCTTCAATATAATTCTGAGACAGAAAGCTGGGACCTGGAATCCGGTTCTGCTGCTAGT
R_Swi2-9V5	CTGTAAAATACTGATATGACAAAAAAAAACAAAAATAGATAGAAACAACAGACAGGAAGCAAAGAAAAAACTAAAAACATATACCTCGAGGCCAGAAGAC
**Gel shift assay**	
F_*SRE3*	TTATCCAAATATGTTTGTTTGGCCGAT
R_*SRE3*	GGGTAAGAAGAACTTTTATTTATTTATTTGCC
F_*SRE2*	GTTTGTGATTATGCTGTTCAGCATTG
R_*SRE2*	GCGAAGCATATTTCTTGCTAATCTTTTG’
F_*mat1* region	TTTCCAATTATGCTGTTCGTGTCATTC
R_ *mat1* region	AGAATGCTCTATGGTTGAGGAAGT
FAM-*H1*	CATGGAAACGAACGAGAGAAAACAAAGGAGAAAGACTATACATTTATATTACAAAAAAA
**Multiplex PCR**	
FAM-MT1	AAATAGTGGGTTAGCCGTGAAAGG
MP1	ATCTATCAGGAGATTGGGCAGGTG
MM1	GGGAACCCG CTGATAATTCTTGG
**aPCR**	
*SRE2*_Forward	ACCTTGTGGTTGATTTCACGTT
*SRE2*_Reverse	ACGGACTAACAAGGAAGCGT
*SRE3*_Forward	TGCCAACATAACGATATCATCA
*SRE3*_Reverse	GCCTAGCGATTGATGTCAGTG
*act1*_Forward	CTCAAAGCAAGCGTGGTATTT
*act1*_ Reverse	TCTTTTCCATATCATCCCAGTTG
**Protein Expression**	
pET_Forward	TAACTGGCCATGGCGATATCGG
pET-SUMO_Reverse	CACAGAGAACAGATTGGTGGT
Swi2-FL_Forward	gaacagattggtggtATGAATGTGAACAAAAAGCAGGAG
Swi2-FL_Reverse	cgccatggccagTTATTCCAGGTCCCAGCTTTCTGTC
Swi2S_Forwad	gaacagattggtggtATGCTGAAGAAAAGAGAAATGACGAG
Swi2C_Forward	gaacagattggtggtGACTTTGATGAAACTTGCATCA
Swi2CL_Forward	gaacagattggtggtCTAATTAACAGAATCCGTAACCTTG
Swi2LN_Reverse	cgccatggccagttATTGATCTACACCGTAGGGATTC
Swi2-AT1-A_Forward	AACTTCGAGGAAGGATTGCC
Swi2-AT1-A_Reverse	TCCTTCCTCGAAGTTCAAATCTTTATTAACATACGAATACTTTCTTGCCGCTGCTGCCCTATGCTTCAC

Swi2-AT2-A_Forward	GTTAATAAAGATTTGAACTTCGAGGAAGGATTGCCAAGAAGAA GGGCAGCAGCTACAGGCTGGAGGAAG
Swi2-AT2-A_Reverse	CAAATCTTTATTAACATACGAATACTTTCTTGG
**D-loop assay**	
pBS_Forward	ATCAAGCTTATCGATACCGTCG
pBS_Reverse	ATCGAATTCCTGCAGCCC
*REIII*_Forward	atcgataagcttgatTCAAAACGCAATTCAAACAACATACC
*SRE3*_Reverse	ctgcaggaattcgatGCACACTCTTCGTTTAATACAAATG

```
S.pombe          MNVNKKQESIPVNTGSESISSNDNERFEQGKGVGSNLGSHFFEPVEYYYSDGKPMNQTEA   60
S.octosporus     ------------------------------------MRVHEFR-------------GSEA   11
S.cryophilus     ------------------------------------MKIHEIP-------------RSEA   11
S.pombe_Sfr1     ------------------------------------------------------------   0

S.pombe          SQMKGTF--SRDFSLNEMNNEFITDSFFCTTTPDPKTESPSFVKYNAHCDDHPEISGHVS   118
S.octosporus     NAVNGENYNL-------KESDFLNGTCDESSS----------------------------   36
S.cryophilus     NTVKGKKHISTGFGIE-SDSSLLNDSSIQNSS----------------------------   42
S.pombe_Sfr1     ------------------------------------------------------------   0

S.pombe          SNDKDFAYFEDKSENQPLVTLPNENNQVIEPLSSQSCKSQLSTQNYSESDFGWNQLCDLD   178
S.octosporus     --------------RKPLSYKPAS-DSYEEPSLQDVCSESIKETGRRIKGISRNRVCPKE   81
S.cryophilus     --------------VQPSSRKPLS-DDFNEPLLTNIPSESFCEPEKRGRSFARNPCSSGK   87
S.pombe_Sfr1     ------------------------------------------------------------   0

                                                          Swi2S    AT-hook1
S.pombe          PIFK---------------SLAFTDDTNLFPAFADSEAALIMLKKREMTRVKHRAGRPRK   223
S.octosporus     PANGDNDRCEG-CLPNERLAANASHLESHLGNLNIPKGYDCMVQPEKIQREKRKAGRPKK   140
S.cryophilus     GPNGDNDQGDAYALENENLSIQPSCLENQLDKFDVSEGHKQGLPKTKIQKEKRKAGRPKK   147
S.pombe_Sfr1     ------------------------------------------------------------   0

                                  AT-hook2
                                                                  Swi6 binding
S.pombe          YSYVNKDLNF-EEGLPRRRGRPTGWRKYPEKEEIYPTLSPR--IKKPRKVFDAVVIPVTI   280
S.octosporus     LSYVAEDDSS-NSSHKRKRGRPKGWRKNPERDVYIEMIDAPCEKRNPKSVFDAVVVPLNN   199
S.cryophilus     LSYVAEDILSEGLSHKRRRGRPRGWRKHPERDVCLEMIDAPVEKHNPKSVFEAVVVPLNS   207
S.pombe_Sfr1     ------------------------------------------------------------   0

S.pombe          KPSIYTEPS-----------LPHHIDWNNGCSE----EFDFEPSREDEDFPDLTSDSTGQ   325
S.octosporus     CKETSKEDSDNEALRNGIQSETNDHTWDINADAVVKPDSNSSDYEMEEPVPGLTFESMTT   259
S.cryophilus     VKNS---------LKQEIANEPNDRTWDFEHHSGMNTDVNMQDGELEDSVPGLTFDS-SI   257
S.pombe_Sfr1     ------------------------------------------------------------   0

S.pombe          DPLSSE------PTIFDISPLPSDMEPTFSENSLITINKMAIEERKQSRRLEQPLAESQH   379
S.octosporus     GPSSSDLPSSGEPLNIAQSDSESETNFGFSEDDFAALDKI--------------------   299
S.cryophilus     GPSSCDLPNNNESLNVTKSDPDSDTNFSFSDDDFAVLDKI--------------------   297
S.pombe_Sfr1     ------------------------------------------------------------   0

                    Swi2N      Swi2C
S.pombe          QEDLLNPYGVDQDFDETCINESEQQATHIPNSSIKF------NYDYTPPKSATSH-KHKR   432
S.octosporus     ----------EQDFEAHDFVQSN---QFSSPDSISRKILVCEQNETSPQKGANEVMKLSR   346
S.cryophilus     ----------EHEFEIHDTDQPK---STSPSSSPSHRITLCENNEALPQKSVTEIKEIQK   344
S.pombe_Sfr1     -----------------------------------------------------------M   1

S.pombe          VDLLASRKENVWTGLYGKVQTEMVSNRGEAVLNENNSKNRTNVTKPNSYKNSVLS-IGNN   491
S.octosporus     GTTVGNRKENNN------------------IFNTN-TGGKANEIG-MRIP--STS-SGSS   383
S.cryophilus     NNTLNSRKENNN------------------IFQSS-SKAKAHHAG-MRFHVRPTF-NGRS   383
S.pombe_Sfr1     SQTINSELNENATS---QCKEDLK-----VSLSESDLR---DSQGQLGIENPPKCNNSGN   50
                    : ..  ::                     :. .      .               . .

S.pombe          HSNHSNIIKPNTYKN--TILSNENNTPNYSNVCLSTSLINRSLPSLKSTMHPGVNKDLIT   549
S.octosporus     KFEKKSFGNPANLKYPNFLKSPRD-ST---------------------KFLNTRTKPSVS   421
S.cryophilus     NLEAKSIGKPSNFKYPSFLKSSRN-NE---------------------NTSSARNKPLVR   421
S.pombe_Sfr1     HSDNLG-----------FIEQSETVHPENE-KAL--------TPDLRDT--KIHTSLPIT   88
                 : :  .            : . .                         .     ..  :

S.pombe          RPFKNV-NKMSVRKALIKPFHPPISKISRTRLTVSSPERLYCAK----------------   592
S.octosporus     NLFASKNSNYRTKQALFKPFRPPFKKDAPFNVIDEETKPTHASS----------------   465
S.cryophilus     SPFAQNNSGDRIKQTLSKPFRPPLKKDALHNIIEEGTEPESGPR----------------   465
S.pombe_Sfr1     TPFSKK-RAREAKNILLKPFKSPLRQTASPQVADTNLKPSLAVTNLNSDETNTSSEPVTS   147
                   * .       :: * ***: *: : :  .:     :
```

```
                                                       Swi2CS
                                                     Swi5 binding
S.pombe          -------------------PISMAT--APSETDSKLINRIRNLELEIGGLKEQLSVVELAL     632
S.octosporus     -------------------SSRMMHLDDANLTNDGHSSVVSRLQSEVSNLQDQVSIVELAY     507
S.cryophilus     -------------------PRRLHHDNV-----AAGSLTINKLQSEISTLQDQISVVELAN     502
S.pombe_Sfr1     PLRTTPNSIKRQKRLFKSPISNCLNPKSDPEITQLLSRRLKLEKEVRNLQEQLITAETAR     207
                                                         .*: *:  *::*:  .* *


S.pombe          DTDKNSKQIQVVERKIQNWRKSAQLAVEVLFPVFSLKFTTMLQEVPQS------VLRTSA     686
S.octosporus     DLENNQEDEMGLLEKIQRWRRSAQLAVEVLFPVFSLKFTTMLQEIPES------VLPSVV     556
S.cryophilus     DLENTQEDEVKLYENIQRWRRSAQLAVEVLFPVFSLKFTTMLQEVPES------VLPSVV     561
S.pombe_Sfr1     KVEAKNED-KDLQTLIQKWKNAAQQAAEVLFKPMAERIRLA-GGVTQSFRIEEGENKGQI     265
                 . : ..::   :   **.*:.:** *.****  :: ::      : :*

S.pombe          NDLRTKPCSIGTYLEQLQIPFHLLQYNSETESWDLE-     722
S.octosporus     DDLRSKPCNIGTYLEQLNIPFSLLNYNPENDSWGDET     598
S.cryophilus     DNLRSKPCNIGTFLEQLDIPFSLLNYNPDSDSWGDEA     593
S.pombe_Sfr1     QEVR-TEFTMSMFLNQFGVPVHLMSFDEENGDWKS--     299
                 :::* .  .:. :*:*: :*. *:.:: :. .*
```

Supplementary Figure S1. Sequence alignment of Swi2 proteins in fission yeast.
Swi2 sequences from *S. pombe*, *S. octosporus*, and *S. cryophilus* were aligned with Clustal Omega.

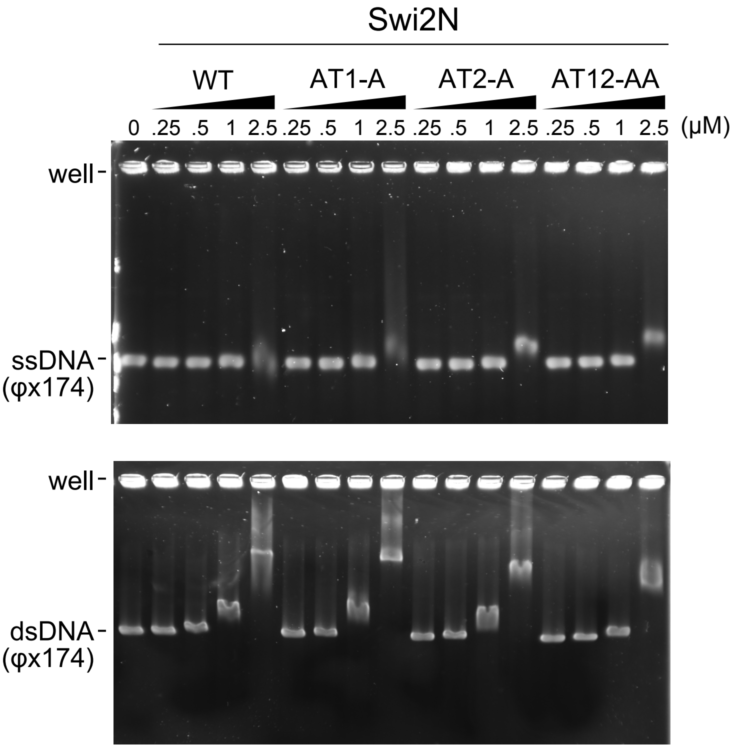

Supplementary Figure S2. EMSA of Swi2N

EMSA of Swi2N derivatives with a cssDNA (ΦX174 viral DNA) and a circular covalently closed DNA (ΦX174 replicative form DNA).

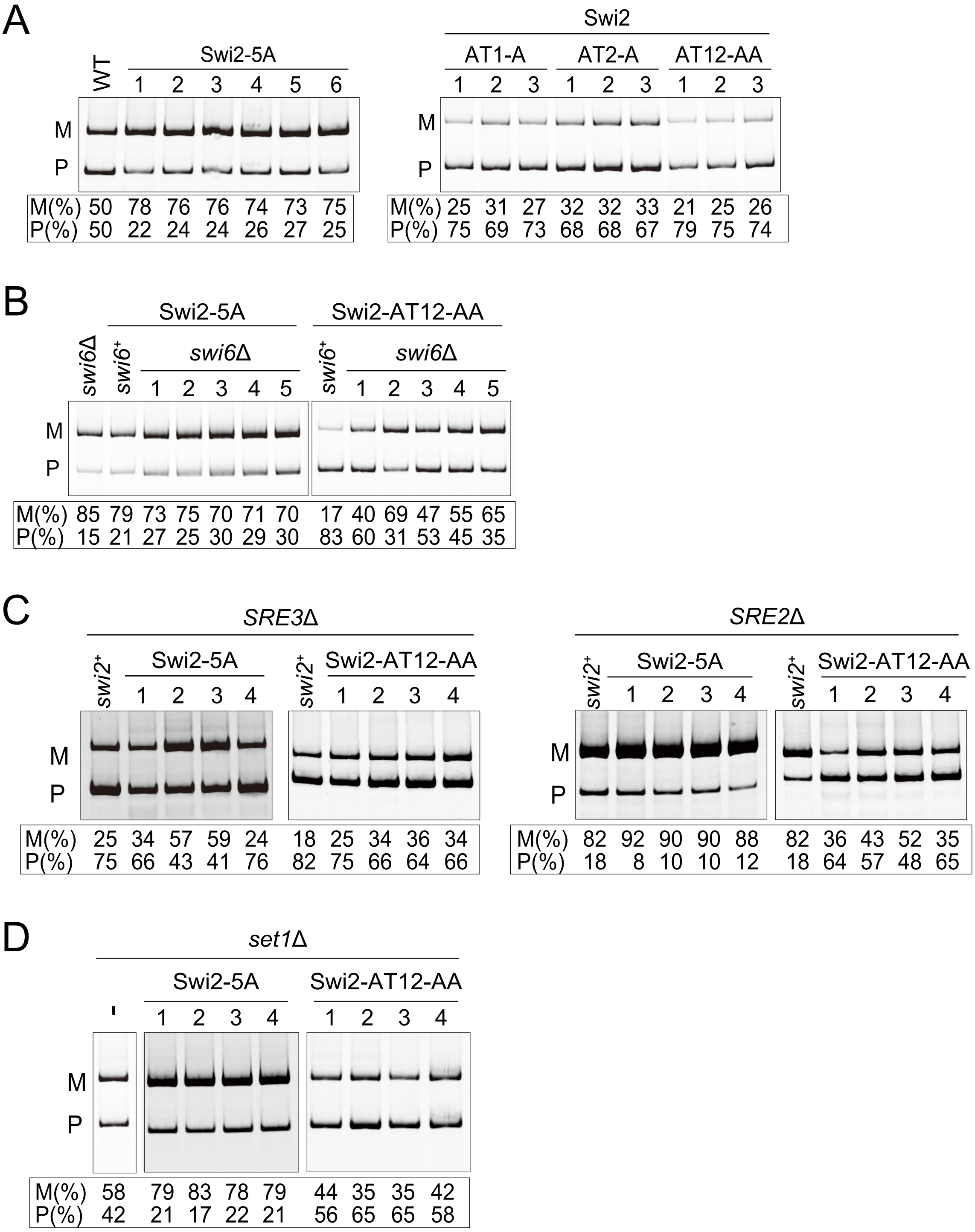

Supplementary Figure S3. Multiplex PCR analysis of Swi2 mutant cells

(A–D) Gels of multiplex PCR analysis of the Swi2 mutants shown in Figures 4B, 4C, 5B, and 5C. The *mat1-P* and *mat1-M* band intensities were measured, and the percentage of the P or M band intensity was calculated as P/(P+M)×100 or M/(P+M)×100 for each single Swi2 mutant (5A, AT1-A, AT2-A, and AT12-AA) (A), the Swi2-5A *swi6*Δ and Swi2-AT12-AA *swi6Δ* mutant strains (B), the Swi2 mutants combined with deletion of *SRE* elements, *SRE2*Δ or *SRE3*Δ (C), and Swi2-5A *set1*Δ and Swi2-AT12-AA *set1*Δ (D).
